# Perioperative statin administration with decreased risk of postoperative atrial fibrillation, but not acute kidney injury or myocardial infarction: A meta-analysis

**DOI:** 10.1038/s41598-017-10600-x

**Published:** 2017-08-30

**Authors:** Li Zhen-Han, Shi Rui, Chen Dan, Zhou Xiao-Li, Wu Qing-Chen, Feng Bo

**Affiliations:** 1 Department of Metabolism and Endocrinology, Shanghai East Hospital, Tongji University School of Medicine, Shanghai, 200120 China; 2 Department of Cardiology, the First Affiliated Hospital of Chongqing Medical University, Chongqing, 400016 China; 3 Department of Cardiothoracic Surgery, the First Affiliated Hospital of Chongqing Medical University, Chongqing, 400016 China

## Abstract

A controversy effect of perioperative statin use for preventing postoperative atrial fibrillation (POAF) and acute kidney injury (AKI) after cardiac surgery still remains. We thus performed current systematic review and meta-analysis to comprehensively evaluate effects of statin in cardiac surgery. 22 RCTs involving 5243 patients were included. Meta-analysis of 18 randomized controlled trials with 3995 participants suggested that perioperative statin use could decrease the risk of POAF (relative risk [RR] 0.69, 95%CI 0.56 to 0.86, *P* = 0.001), with a moderate heterogeneity (*I* ^2^ = 65.7%, *P*_*H*_ < 0.001). And the beneficial effect was found only in patients receiving coronary artery bypass graft (CABG), but not in patients undergoing valve surgery. However, perioperative statin use was not associated with lower risks of AKI (RR 0.98, 95%CI 0.70 to 1.35, *P* = 0.884, *I* ^2^ = 33.9%, *P*_*H*_ = 0.157) or myocardial infarction (MI) (RR 0.84, 95%CI 0.58 to 1.23, *P* = 0.380, *I* ^2^ = 0%, *P*_*H*_ = 0.765), and even an increased trend of AKI was observed in patients with valve surgery. Perioperative statin use could decrease the inflammation response with no impact on clinical outcomes. In conclusion, perioperative statin use is useful in preventing POAF, particularly in patients with CABG, and ameliorate inflammation, while it has no effect on AKI and MI after cardiac surgery.

## Introduction

Despite advanced protection of cardiopulmonary bypass (CPB) and other techniques supported during cardiac surgery, the major post-operation complications are still like Pandora’s Box, contributing to the substantial mortality and morbidity and increasing medical costs^1, 2^. Currently, researches demonstrated that these complications were mainly driven by post-perfusion syndrome, oxidative stress and release of inflammation cytokines after cardiac surgery^3, 4^. Though as transient complications, the indisputable fact is that postoperative atrial fibrillation (POAF) and acute kidney injury (AKI), the most frequent complications after cardiac surgery, are independent risk factors related to poor prognosis in patients received cardiac surgery^5, 6^.

Observational studies, randomized controlled trials (RCTs), and meta-analysis have demonstrated that perioperative statin use could decrease the incidence of POAF and AKI^7–10^, and latest guidelines suggested statins should be administrated in all patients undergoing coronary artery bypass graft (CABG) except for specific contradictions^11^. However, recent studies fail to verify the beneficial effect of statin use in cardiac surgery, and the controversy still exists^12–16^. Though many meta-analyses have been performed, this issue is still fuzziness. Therefore, we further systematically summarized current evidence of RCTs and meta-analyses to provide a comprehensive evaluation and try to answer the following questions in patients without chronic statin use: 1) verify the association between perioperative statin use and POAF, and clarify the impact of related factors on the association; 2) whether statin could decrease the incidence of AKI, and the effect of other related factors on the association between perioperative statin use and AKI after surgery; 3) the effect of perioperative statin use on other clinical outcomes and biochemical indexes.

## Results

### Literature research

**Figure 1:**
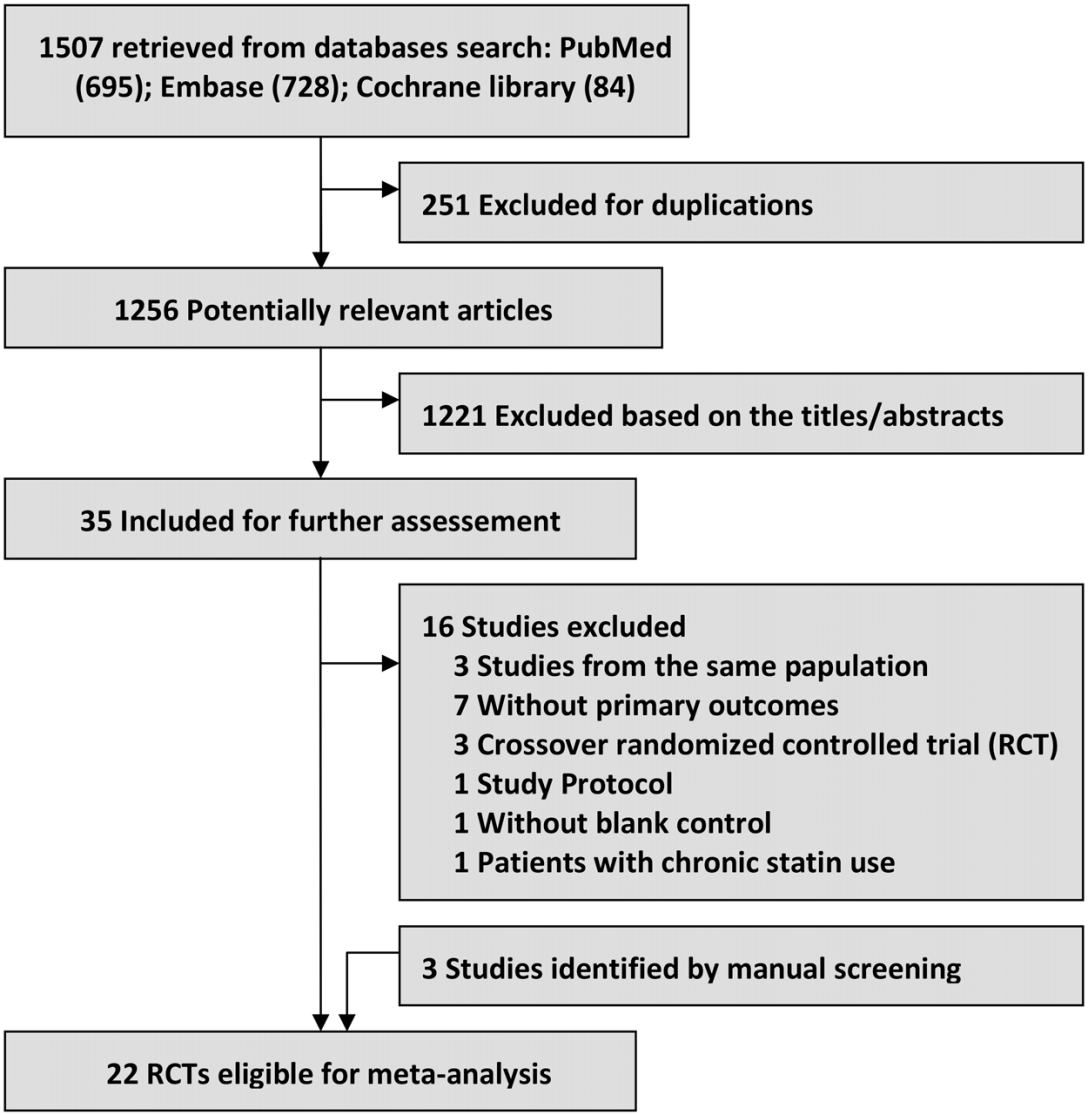
Flow chart of the study selection.

Figure 1 shows the process of literature research and details of selection. 1507 citations were identified after the initial screening. 251 articles were excluded because of duplication, and 1221 were excluded based on the titles or abstracts. 35 remaining studies were further assessed by evaluating the full-text manually, of which 16 studies were excluded owing to the reasons presented in the flow chart. Additionally, three studies were retrieved by manually screening of the reference lists. Finally, 22 independent RCTs were included in current meta-analysis^8, 12–14, 17–34^.

### Study characteristics

The basic clinical and demographic characteristics of the 22 eligible RCTs are listed in Table 1. Overall, our meta-analysis consisted of 5243 patients and 64.0% of them were males, with 2627 patients in the statin group and 2616 in routine medication or placebo group. Among all 22 studies, 18 trials with 3995 participants reported the outcome of POAF, and nine with 3214 patients reported AKI. 14 RCTs enrolled patients undergoing CABG, three RCTs undergoing valve surgery^13, 23, 26^. For studies only involving patients with CABG, five of them were with CPB^17, 21, 24, 25, 32^, three with off-pump^8, 17, 30^, and six with CPB or off-pump^19, 20, 22, 28, 29, 33^. All patients take statins with a different duration ranging from one day to four weeks before the surgery. For statin administration, different statin types (atorvastatin, simvastatin, fluvastatin, rosuvastatin, and pravastatin) and different doses of atorvastatin ranging from 20 to 80 mg were used. For the control group, placebo was used in 15 trials while routine medication alone without statins was administrated in the other seven trials. All the outcomes are presented in supplementary tables **(**Supplementary information, Appendix 1–4). Following the Cochrane risk of bias assessments tool, 15 trials were assessed as unclear risk of bias because of other bias rating as unclear risk and 7 studies were at high risk of bias (Supplementary information, Appendix 5).

**Table 1:** Baseline characteristics of the 22 included RCTs.

**StudyID**	**Country**	**Population(Statin/Control)**			**Surgery type**	**Intervention**	
		**No**.	**Age**	**Man(%)**		**Statin group**	**Control**
Almansob *et al*. 2012^18^	China	68/64	45.5 ± 14.5/41.5 ± 18.7	49.20%	Noncoronary cardiac surgery	Routine medication + Simvastatin: 20 mg/day, 5–7 days before surgery and restart in the second day postoperation	Routine medication without statin
Aydin *et al*. 2015^19^	Turkey	30/30	62.6 ± 10.9/62.4 ± 12.2	39.20%	CABG	Routine medication + Atrovastatin: 40 mg/day, 6 hours after surgery until postoperaive 1 month	Routine medication without statin
Baran *et al*. 2012^20^	Turkey	30/30	60.8 ± 8.6/62.2 ± 8.1	61.70%	CABG	Routine medication + Atrovastatin: 40 mg/day, 14 days before surgery and restart in the first day postoperation	Routine medication + Placebo
Berkan *et al*. 2009^21^	Turkey	23/23	65.4 ± 11.2/67.7 ± 9.6	63.00%	CABG + CPB	Routine medication + Fluvastatin: 80 mg/day, 3 weeks before surgery	Routine medication + Placebo
Billing *et al*. 2016^14^	USA	308/307	66 ± 6.7/67 ± 6.3	69.40%	Cardiac surgery	Routine medication + Atorvastatin: 80 mg/day, 1 day before surgery and 40 mg/d after surgery until discharge	Routine medication + Placebo
Caoris *et al*. 2008^22^	Chile	21/22	68.2 ± 7.2/67.9 ± 7.3	83.70%	CABG	Routine medication + Pravastatin: 40 mg/day, 2 days before surgery and 7 days after surgery with an additional dose of 40 mg at 1 hour after surgery	Routine medication without statin
Carascal 2016^23^	Spain	47/43	67.4 ± 11.2; /65.5 ± 12.0	65.56%	Valve surgery	Routine medication + atrovastatin 40 mg/d 7days before surgery until lasting 7d after surgery	Routine medication without statin
Chello *et al*. 2006^24^	Italy	20/20	65.7 ± 7.7/63.7 ± 7.1	77.50%	CABG + CPB	Routine medication + Atrovastatin: 20 mg/day, 3 weeks before surgery	Routine medication + Placebo
Chritanson *et al*. 1999^25^	Belgium	40/37	62.7 ± 11.3/64.1 ± 10.8	79.50%	CABG + CPB	Routine medication + Simvastatin: 20 mg/day, 4 weeks before surgery	Routine medication without statin
Dehghani *et al*. 2015^26^	Iran	29/29	54 ± 6.5/45 ± 6.5	32.80%	Valve surgery + CPB	Routine medication + Atrovastatin: 40 mg/day, 3 days before and 5 days after surgery	Routine medication + Placebo
Ji *et al*. 2009^27^	China	71/69	65 ± 6/66 ± 9	69.30%	CABG + off-pump	Routine medication + Atrovastatin: 20 mg/day, 7 days before surgery	Routine medication + Placebo
Makuucdi *et al*. 2005^28^	Japan	152/151	59.6 ± 6.5/58.2 ± 7.3	84.20%	CABG	Routine medication + Pravastatin: 10–20 mg/day	Routine medication without statin
Melina *et al*. 2009^30^	Italy	315/317	NR	NR	CABG + off-pump	Routine medication + Atrovastatin: 40 mg/day before surgery	Routine medication + Placebo
Mannacio *et al*. 2008^29^	Italy	100/100	61.3 ± 9.2/59.3 ± 8.4	72.50%	CABG	Routine medication + Rosuvastatin: 20 mg/day, 7days before surgery	Routine medication + Placebo
Park *et al*. 2016^13^	Korea	100/100	58 ± 12/58 ± 14	49.50%	Valve surgery	Routine medication + Atorvastatin: 80 mg/day, 1 day before surgery and 40 mg 2 after surgery, with 80 mg the day of surgery	Routine medication + Placebo
Patti *et al*. 2006^31^	Italy	101/99	65.5 ± 8.8/67.3 ± 8.1	73.50%	Cardiac surgery + CPB	Routine medication + Atrovastatin: 40 mg/day, 7days before surgery	Routine medication + Placebo
Prowle *et al*. 2012^12^	Australia	50/50	69.0 ± 11.1/67.3 ± 10.8	70%	Cardiac surgery + CPB	Routine medication + Atrovastatin: 40 mg/day, 1 day before surgery and 3 days after surgery	Routine medication + Placebo
Song *et al*. 2008^8^	Korea	62/62	61.7 ± 9.9/64.0 ± 9.2	65.30%	CABG + off-pump	Routine medication + Atrovastatin: 30 mg/day, 3 days before surgery and 30 days after surgery	Routine medication + Placebo
Sun *et al*. 2011^32^	China	49/51	64 ± 7/65 ± 8	67%	CABG + CPB	Routine medication + Atrovastatin: 20 mg/day, 7 days before surgery	Routine medication + Placebo
Tamayo *et al*. 2009^17^	Spain	22/22	67.7 ± 7.3/68.0 ± 6.9	79.50%	CABG + CPB	Routine medication + Simvastatin: 20 mg/day, 3 weeks before surgery	Routine medication without statin
Vukovic *et al*. 2011^33^	Serbia	29/28	61.3 ± 7.7/61.8 ± 7.4	84.20%	CABG	Routine medication + Atorvastatin :20 mg/day, 3 weeks before surgery	Routine medication + Placebo
Zheng *et al*. 2016^34^	China	960/962	59.3 ± 9.4/59.5 ± 9.5	79.20%	CABG + Valve surgery	Routine medication + Rosuvastatin: 20 mg/day, 8 days before surgrey and 5 days after surgery	Routine medication + Placebo

RCTs, randomized controlled trials; CABG, Coronary Artery Bypass Grafting; CPB, Cardiopulmonary Bypass;

### Primary outcomes

**Figure 2:**
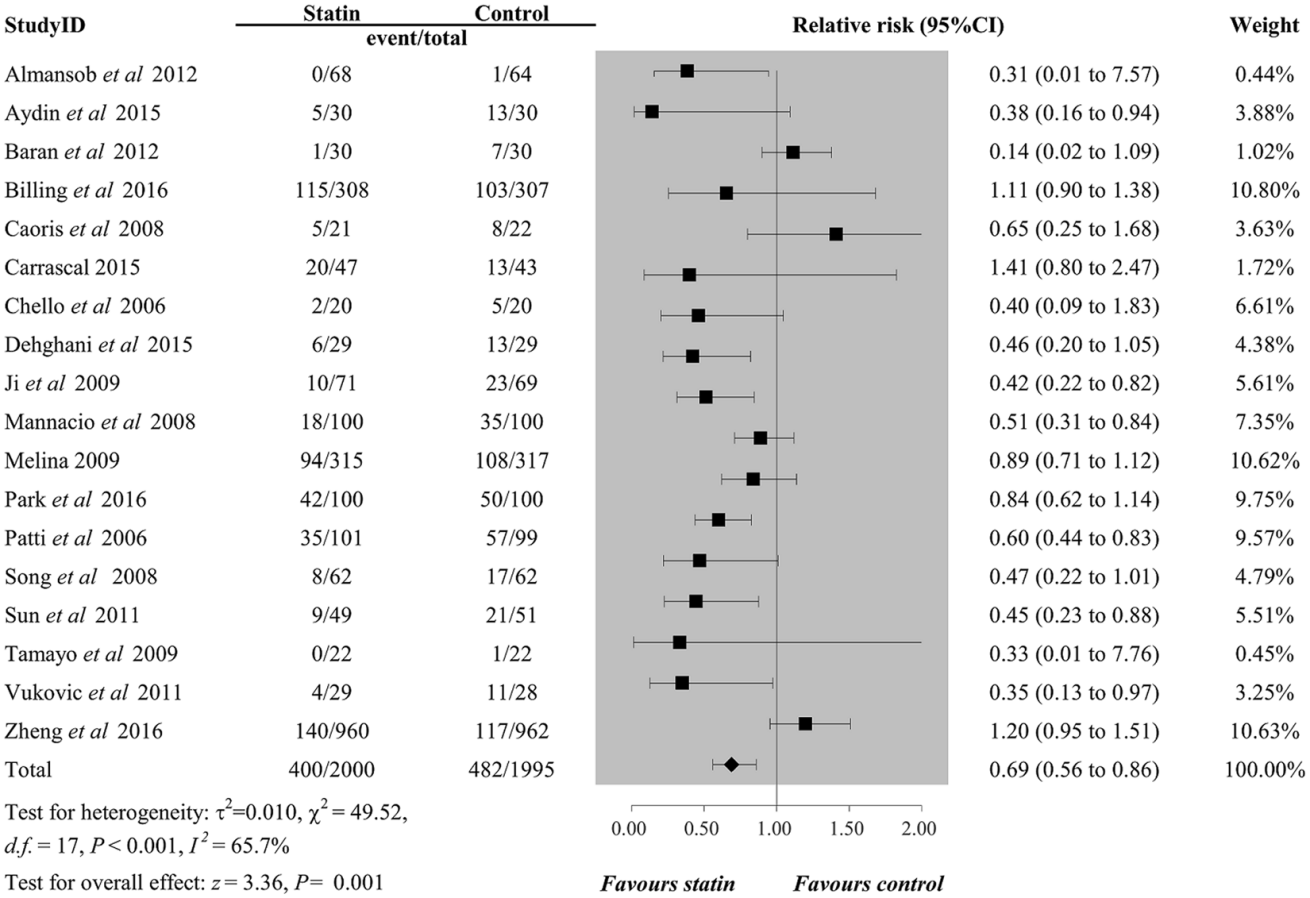
Forest plots for the meta-analysis of the incidence of POAF. POAF, postoperative atrial fibrillation.

**Figure 3:**
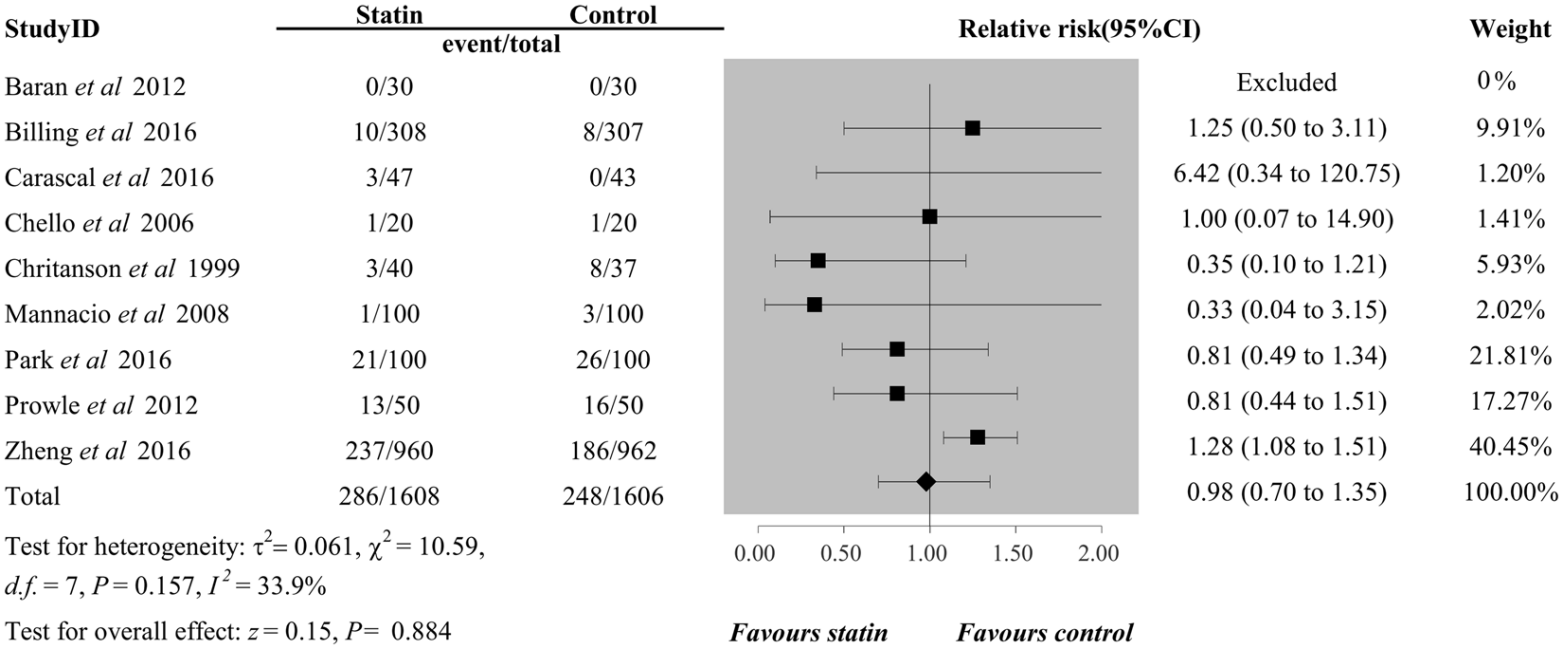
Forest plots for the meta-analysis of the incidence of AKI. AKI, acute kidney injury.

**Figure 4:**
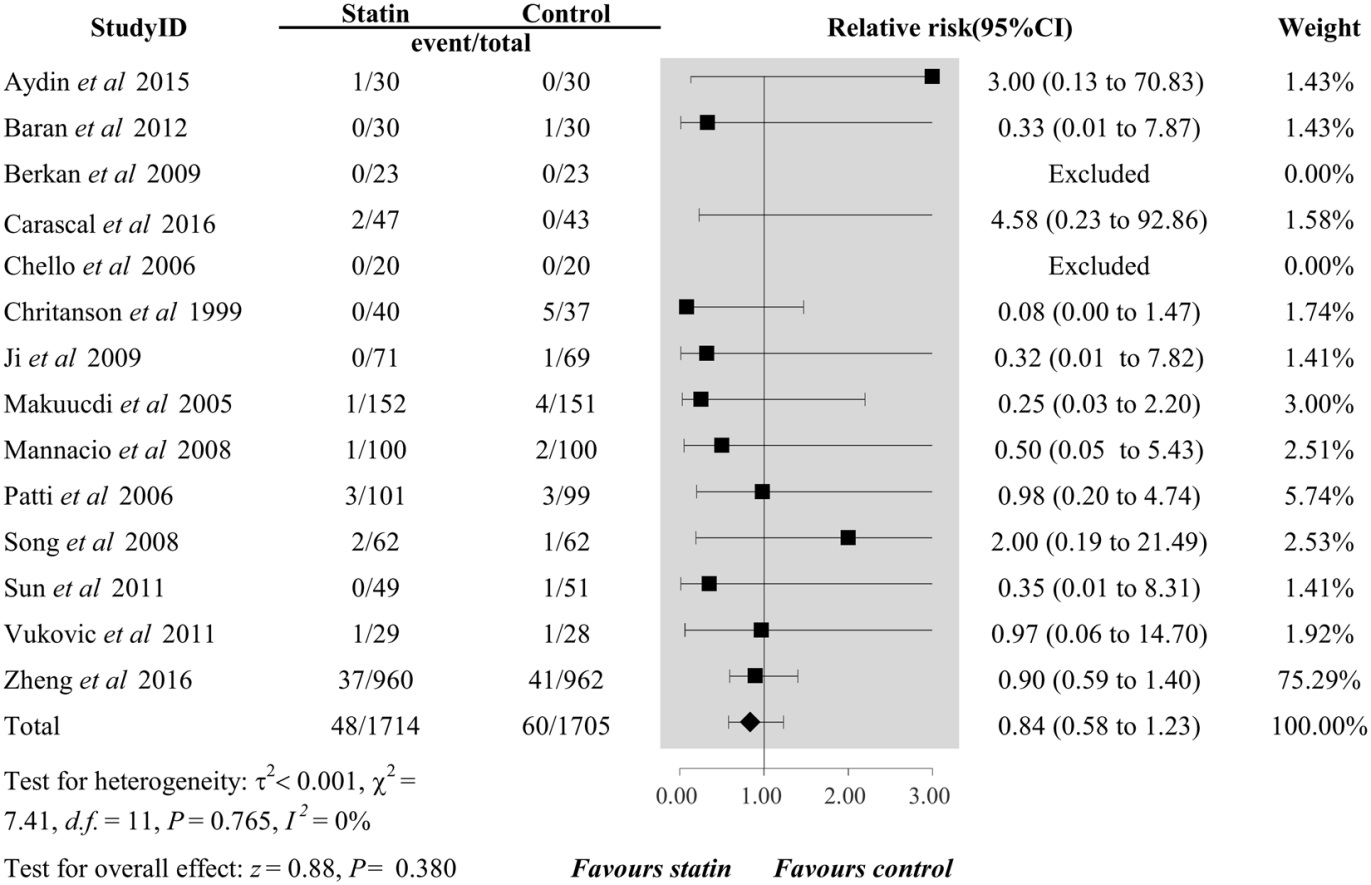
Forest plots for the meta-analysis of the incidence of MI. MI, myocardial infarction.

Using random-effects model, the overall results of 18 trials consist of 3995 participants showed that perioperative statin use significantly decreased the risk of POAF (relative risk [RR] 0.69, 95% confidence intervals [CI] 0.56 to 0.86, *P* = 0.001, Fig. 2) with a significant high heterogeneity (*I* ^2^ = 65.7%, *P*_*H*_ < 0.001), and combined results of nine trials involving 3214 patients failed to show a protective effect of perioperative statin use for preventing the occurrence of AKI postoperatively (RR 0.98, 95%CI 0.70 to 1.35, *P* = 0.884, Fig. 3), with a low heterogeneity (*I* ^2^ = 33.9%, *P*_*H*_ = 0.157). Additionally, perioperative statin use was not associated with decreased risk of MI (RR 0.84, 95%CI 0.58 to 1.23, *P* = 0.380, *I* ^2^ = 0%, *P*_*H*_ = 0.765, Fig. 4).

### Subgroup analysis

**Figure 5:**
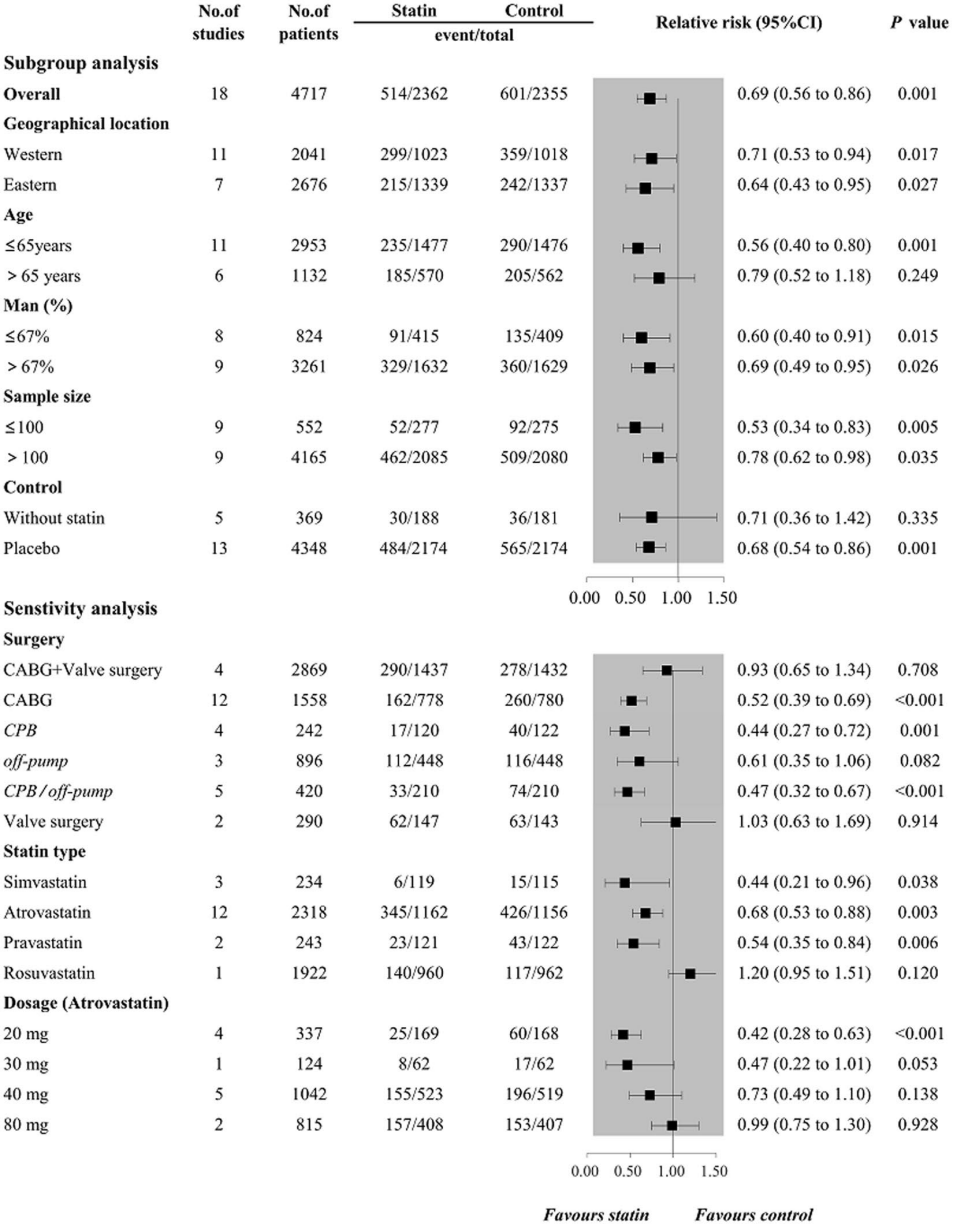
Forest plots for subgroup and sensitivity analyses of the incidence of POAF. POAF, postoperative atrial fibrillation.

**Figure 6:**
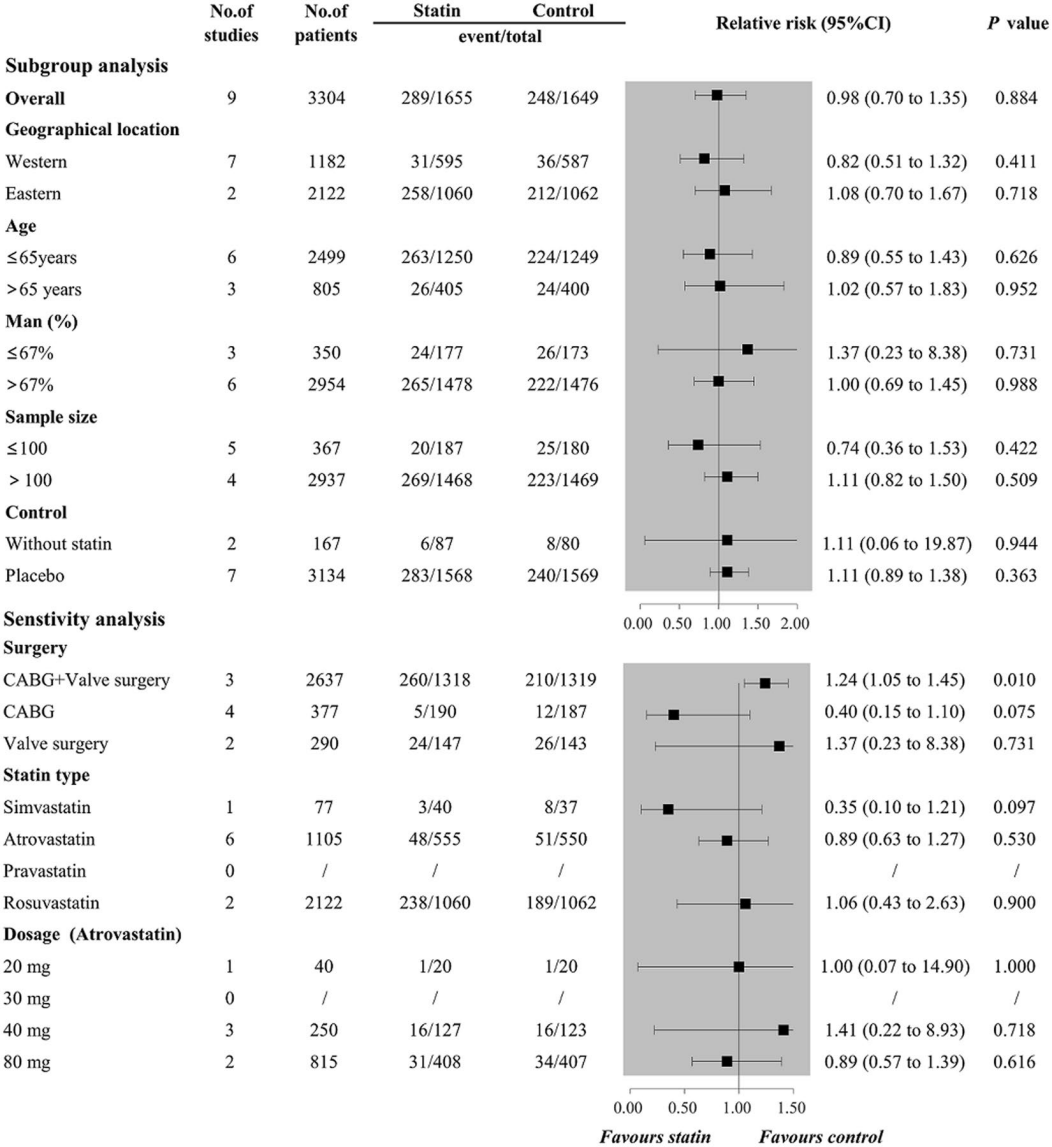
Forest plots for subgroup and sensitivity analyses of the incidence of AKI. AKI, acute kidney injury.

**Figure 7:**
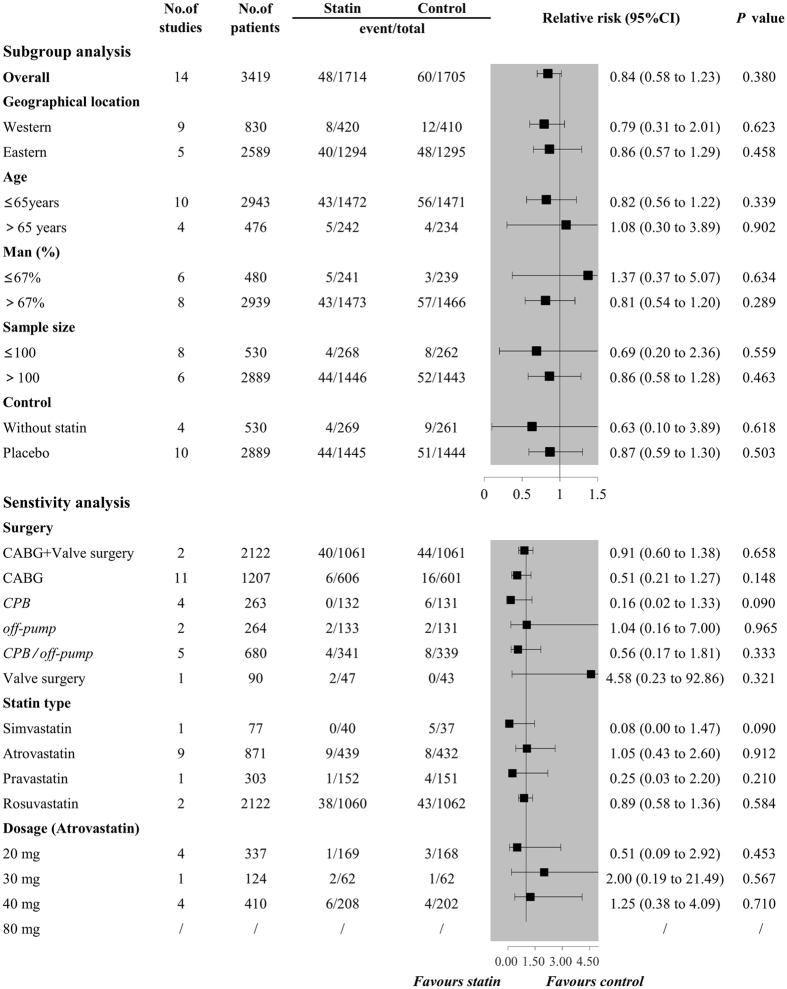
Forest plots for subgroup and sensitivity analyses of the incidence of MI. MI, myocardial infarction.

The results of the subgroup analysis for POAF are shown in Fig. 5. The results remained consistent with the overall estimate when stratified by geographical location, age, the proportion of man, sample size, and control, while results of subgroup analyses were inconsistent when subgrouping by age and control. For the outcomes of AKI and MI, results of subgroup analyses remained consistently with the overall estimate in all stratification factors, showing that no protective effect of perioperative statin use on the occurrence of AKI (Fig. 6) and MI (Fig. 7).

### Sensitivity analysis

Sensitivity analysis for POAF is shown in Fig. 5. According to the types of surgery, perioperative statin use could decrease the risk of POAF in patients with CABG (RR 0.52, 95%CI 0.39 to 0.69), including patients undergoing CABG with CPB (RR 0.44, 95%CI 0.27 to 0.72), but not in patients with valve surgery (RR 1.03 95%CI 0.63 to 1.69). Results showed that perioperative rosuvastatin administration was even associated with increased risk trend of POAF (RR 1.20 95% CI 0.95 to 1.51, *P* = 0.120), while other statins could significantly reduce the incidence of POAF. Additionally, sensitivity analysis by omitting one study in each turn showed that no single study could substantially alter the pooled effect with RRs ranging from 0.65 (95%CI 0.52 to 0.82) to 0.72 (95%CI 0.58 to 0.89).

Sensitivity analyses for AKI and MI are shown in Figs 6 and 7. All revealed that perioperative statin use failed to decrease the incidence of AKI or MI. Additionally, sensitivity analysis by omitting one study in each turn also confirmed the null association, with pooled RRs ranging from 0.82 (95%CI 0.58 to 1.14) to 1.10 (95%CI 0.87 to 1.40) for AKI and pooled RRs ranging from 0.69 (95%CI 0.32 to 1.46) to 0.88 (95%CI 0.60 to 1.29) for MI.

### Secondary outcomes of clinical ones

The pooled result suggested that perioperative statin use was not significantly associated with decreased mortality (RR 1.13, 95%CI 0.56 to 2.27, *P* = 0.740), duration of mechanical ventilation (MV) (standard mean difference [SMD] −0.01, 95%CI −0.44 to 0.42, *P* = 0.967), duration of intensive care unit (ICU) stay (SMD 0, 95%CI −0.12 to 0.12, *P* = 0.987), or hospital length of stay (HLOS) (SMD −0.18, 95%CI −0.37 to 0, *P* = 0.051). The results of secondary outcomes of clinical ones are shown in Table 2.

**Table 2:** Pooled effect sizes of secondary outcomes.

Outcomes	No. of studies	No. of patients	*I* ^2^	*P*_*H*_	Effect size (95%CI)	*P* value
					**SMD (95%CI)**	
MV	8	765	86.9%	<0.001	−0.01 (−0.44 to 0.42)	0.967
ICU length of stay	14	3630	45.7%	0.032	0 (−0.12 to 0.12)	0.987
HOLS	14	3217	74.8%	<0.001	−0.18 (−0.37 to 0)	0.051
					**RR (95%CI)**	
Mortality	12	3725	0.0%	0.428	1.13 (0.56 to 2.27)	0.740

MV, Mechanical ventilation; ICU, Intensive care unit; HLOS, Hospital length of stay; RR, Relative risk; SMD, Standard mean difference; CI, Confidential interval

### Secondary outcomes of biochemical indexes

**Figure 8:**
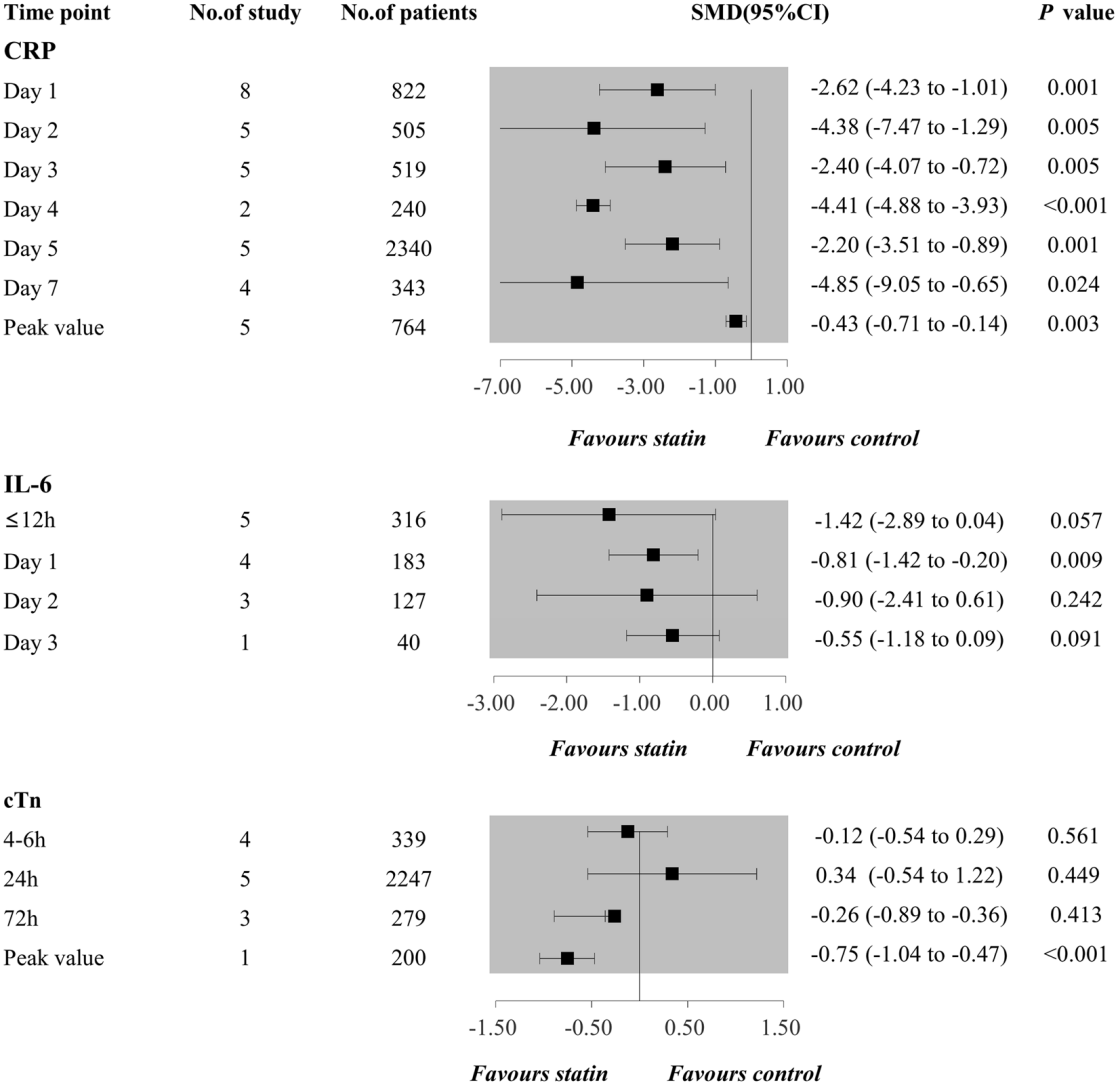
Forest plots for the meta-analysis of the biochemical indexes (CRP, IL-6, cTn) postoperatively. CRP, C-reaction protein; IL-6, interleukin-6; cTn, cardiac troponin.

The combined resulted showed that perioperative statin use could significantly reduce the peak concentration of C-reactive protein (CRP) (SMD −0.43 95%CI −0.71 to −0.14, *P* = 0.003; Fig. 8) and concentration of CRP from the first to seventh day postoperatively (SMDs ranging from −4.85 to −2.20; Fig. 8). Additionally, perioperative statin use could decrease peak concentration of cardiac troponin (cTn), cTn in 72 h after surgery, and IL-6 in the first day after surgery, as shown in Fig. 8. Though it failed to lower IL-6 and cTn in other time points, a decreased trend could be observed in all of them.

## Discussion

Our meta-analysis, included 22 RCTs consisting of 5243 participants, found that perioperative statin use could significantly decrease the incidence of POAF, and the beneficial effect was associated with surgery type, statin type and statin doses. While it failed to reduce the incidence of AKI and MI, and the null association remained consistent in most subgroup and sensitivity analyses. Even that perioperative statin use was associated with increased risk of AKI in patients receiving CABG or valve surgery. Perioperative statin use was not associated with decreases of HLOS, mortality, duration of MV, and ICU length of stay. However, it could decrease postoperative inflammation response of CRP and IL-6 and myocardial injury marker of cTn.

The mechanism of statin decreasing POAF after cardiac surgery still remains unknown, and following points have been identified. Statin, as a drug of lowering cholestenone and stabilizing atherosclerosis plaques in coronary artery disease, has been reported to contribute toward the stabilization of transmembrane ion channel, modification the extracellular matrix remodeling, and has effect of anti-inflammation and anti-oxidant. The effect of anti-inflammation had been confirmed by decreased CRP and IL-6 after surgery in the current study, while anti-inflammation and anti-oxidant were involved in the development of both POAF and AKI. Therefore, the decreased incidence of POAF might be owing to the anti-inflammation and anti-oxidant of statin. While the non-beneficial effect of statin on AKI could be explained by the very vulnerability of kidney and severe injury beyond the benefit from anti-inflammation and anti-oxidant of statin. Additionally, the high susceptivity of POAF on inflammation and oxidization might also be involved in the inconsistent finding between AKI and POAF. In our study, statin seemed not to exert a protective effect, and even increase the risk of AKI when rosuvastatin used. The underlying mechanism might as follows: Firstly, the duration of the assigned regimen was not sufficient since it was up to 8 days before the surgery, whereas it was reported that statins need 14 days to exert effect^35^. Secondly, the study finding of increased AKI risk of rosuvastatin was conducted in Asian population, while studies have demonstrated that Asian patients were more likely to have side effects than European patients at the same dose of statin regimen^36^. Thirdly, contrast agent was always used before cardiac surgery to assess the coronary artery disease, and contrast-induced injury was also involved in kidney injury independent of cardiac surgery.

In our study, subgroup analyses for POAF remained robust and stable for most stratified factors, while it was inconsistent in patients older than 65, control of no statin, valve surgery and off-pump CABG, rosuvastatin, dose of atrovastatin more than 30 mg. The following explanations might persuade the inconsistence of null association: Patient with older age were more frequently with other medication uses, such as beta-blocker, NSAID, glucocorticoid, insulin, antiplatelet, anticoagulant, calcium-channel blocker, ACE inhibitor or ARB, and diuretic agent. Many of the current medications have been verified to have preventive effects in prevention of POAF, and this effect could weaken the benefit of statins. The inconsistence between controls could be explained by the placebo effect and methodology of allocation concealment. Valvular diseases are mostly related to the ageing process due to calcification of valves but not the inflammation responses or lipid deposition. Therefore, patients with valve surgery had a relatively lower cardiovascular risk and thus failed to benefit from lipid-lowering and anti-atherosclerosis effect of statin use like CABG. While for patients received off-pump CABG, off-pump surgery itself could reduce the incidence of POAF via decreasing inflammatory response, and thus the trend of lowering POAF in patients with off-pump CABG was not as strong as in patients with on-pump surgery. Null association of rosuvastatin in our subgroup analysis highlighted the effect of different statin type in prevention of POAF. While differences in dosage of statin could be attributed to the fact that high-dose statin increasing the side-effect. Subgroup and sensitivity analyses revealed that statin was not associated with lower risk of AKI, and even increased the incidence of AKI in patient undergoing valve surgery or CABG. And this increasing effect was mainly driven by the study by Zheng, which was only one that found the significant increased risk of AKI in the included subgroup. Additionally, rosuvastatin, used in the study by Zheng, had been demonstrated to be more susceptible to renal toxicity, and above than 40 mg were avoided according to FDA and 20 mg according to CFDA owing to its renal toxicity. Based on above, our subgroup and sensitivity analysis suggested that perioperative statin use is useful in preventing POAF after cardiac surgery, especially effective in patients with CABG. While statin seems to have a harmful effect on AKI after cardiac surgery, especially in rosuvastatin use. Considering limitations of subgroup and sensitivity analysis, this conclusion should be interpreted cautiously, and further investigations are needed.

Current meta-analysis shares the similar results with the previous systematic review and meta-analyses^7, 37–40^, and the recent ones are summarized in Table 3. Though consistent, the current meta-analysis generally concurs and further extends the finding of previous meta-analysis in several important ways. Firstly, our meta-analysis reinforced the earlier results by including additional studies. Additionally, the current study also evaluates the effect of statin on the chemical index of CRP, IL-6, and cTn after cardiac surgery, while none of the previous ones focused. Moreover, exclusion of each single study, subgroup and sensitivity analyses according to age, sample, statin type, dosages of statin, and surgery type, were performed to test the robustness to our main finding, and to clarify the potential role of them in heterogeneity.

**Table 3:** Comparison with previous meta-analyses.

Study	No. of trials	Primary Outcome	Secondary outcomes	Chemical indexes	Main results (OR/RR)
Patti *et al*.^7^	11 RCTs	POAF	Myocardial injury, MACE, mortality, stroke	CRP	POAF: 0.41 (0.31 to 0.54)
Putzu *et al*.^37^	23 RCTs (including cross-over trial)	AKI, POAF, MI, stroke, infection	Mortality	NR	POAF: 0.80 (0.70 to 0.91) AKI: 1.18 (0.99 to 1.41)
Rezaei *et al*.^38^	12 RCTs (including cross-over trial)	POAF	Duration of MV, ICUstay, HLOS	CRP	POAF: 0.50 (0.41 to 0.61)
Xiong *et al*.^39^	9 RCTs	AKI, RRT	ICUstay, HLOS	Scr, CRP	AKI: 1.12 (0.97 to 1.29)
Yuan *et al*.^40^	20 RCTs (including cross-over trial)	POAF, AKI, mortality	MI, stroke, ICU stay, HLOS	Scr	POAF: 0.50 (0.34 to 0.73) AKI: 1.01 (0.75 to 1.36)
Current one	22 RCTs	POAF, AKI, MI	Mortality, ICU length of stay, HLOS	CRP, IL-6, cTn at different time	POAF: 0.69 (0.56 to 0.86) AKI: 0.98 (0.70 to 1.35) MI: 0.84, (0.58 to 1.23)

RCTs, randomized controlled trials; POAF, postoperative atrial fibrillation; AKI, acute kidney injury; CRP, C-reaction protein; MI, myocardial infarction; ICU, intensive care unit; HLOS, hospital length of stay; Scr, serum creatinine; MACE, major adverse cardiovascular events; MV, mechanical ventilation.

Our study has several limitations. Firstly, for the incidence of AKI, more than half of the involved patients were from the study by Zheng, and the increased trend of AKI risks was mainly driven by it, with a relatively high weight of 40.45%. Additionally, rosuvastatin, more susceptible to renal toxicity, was only used in the study by Zheng. While, after excluding this study, the result remained still and null, indicating that no effect of perioperative statin on incidence of AKI. Secondly, moderate heterogeneity of *I* ^2^ = 65.7%, which could be attributed to differences in patient characteristics, intervention of statin, and the definition of POAF, was observed for POAF. Nevertheless, the pooled results remain stable in most sensitivity and subgroup analyses, with a low heterogeneity. Finally, potential missing and unpublished data may lead bias to the analysis.

In summary, perioperative statin use is useful in preventing POAF after cardiac surgery, particularly in patients with CABG, and ameliorate inflammation, while it has no effect on AKI and MI after cardiac surgery. Therefore, statin should be given to patient undergoing CABG but not valve surgery for preventing POAF.

## Methods

The guideline for meta-analysis of RCTs–PRISMA (Preferred Reporting Items for Systematic Reviews and Meta-Analyses)^41^ and Cochrane methodology^42^ are followed in our study. Disagreements regarding the study search, study selection, data extraction, and quality assessment were resolved by consensus and the third reviewer as necessary. All analyses were based on previous published studies, thus no ethical approval and patient consent are required.

### Search strategy

Two trained investigators independently searched PubMed, EMbase, and Cochrane library from the inception to Oct 2016. Both free-text terms and subject terms of statin and cardiac surgery were used, and the detailed search strategy was presented in Appendix 6 (Supplementary information, Appendix 6). The reference lists of included studies and relevant reviews were manually searched to avoid missing relevant studies, and conference abstracts were also included.

### Study selection

The study was included if it met the inclusion criteria:(1) Patients undergoing cardiac surgery; (2) Patients treated with perioperative statins but without chronic statin use; (3) Patient treated with placebo or routine medications as comparison; (4) Outcomes included POAF, AKI, and MI; (5) Study design restricted strictly to RCTs. Two reviewers performed independent manual screening of all the articles by firstly the titles/abstracts and secondly the full-texts. Besides, other relevant literatures and references of the included studies were also manually screened.

### Data extraction and outcomes

The relevant basic characteristics of eligible studies, including patients characteristics (author, publication year, country, intervention of statin, control, type of cardiac surgery), and incidence of AKI, incidence of POAF, incidence of MI, mortality, HLOS, duration of MV, ICU length of stay, biochemical indexes of CRP, cTn, and IL-6, were extracted by two reviewers independently using a predefined data extraction sheet. We treated POAF, AKI and MI as primary outcomes. Secondary outcomes included mortality, duration of MV, ICU length of stay, HLOS, and biochemical indexes of changes in CRP, IL-6, and cTn.

### Quality assessment and data analysis

Two researchers independently assessed the quality of each contributing evidence following the recommended Cochrane risk of bias tool^43^ respecting to seven parts of the basis of selection, performance, detection, attrition and reporting bias. Each study was assessed to be of low, unclear or high risk of bias. Continuous variables were expressed as mean ± standard deviation (SD), and data using different parametric were assessed for suitability and converted to mean ± SD by using the corresponded formula^44^. SMDs with 95% CIs and RRs with 95%CIs were used to perform meta-analysis for continuous outcomes and dichotomous outcomes, separately. Statistical heterogeneity of included studies was assessed by *I* ^2^, with rates of low if *I* ^2^ is between 25% and 50%, moderate if *I* ^2^ between 50% and 75%, and high if*I* ^2^ more than 75%^45^. To explore whether the results were altered by study characteristics, subgroup analyses, based on area, sample size, the proportion of male, age, and control group, were performed. Additionally, sensitivity analysis regarding surgery type, type and dosage of statins, was also performed. All meta-analyses were conducted with the random-effects model. Statistical analyses were performed by using Stata 12.0 software (Stata Corp, College Station, TX, USA), and risk of bias was evaluated by using Review Manager Version 5.1 (The Cochrane Collaboration, Software Update, Oxford, UK). A *P* value less than 0.05 suggests a statistical difference.

### Data availability statement

All data generated or analysed during this study are included in this published article and its supplementary information files.

## Electronic supplementary material

Supplementary Information
