# Supplementary material for: Perioperative statin administration with decreased risk of postoperative atrial fibrillation, but not acute kidney injury or myocardial infarction: A meta-analysis
Source: Sci Rep. 2017 Aug 30;7:10091. doi: 10.1038/s41598-017-10600-x (PMC5577099; doi:10.1038/s41598-017-10600-x)
Supplement: Supplementary file 1 — Supplementary Information [file 41598_2017_10600_MOESM1_ESM.pdf]

Perioperative statin administration with decreased risk of postoperative atrial fibrillation, but not acute kidney injury or myocardial infarction: A meta-analysis

Zhen-Han, Li [1#], Rui, Shi [2#], Dan, Chen [3], Xiao-Li, Zhou [2], Qing-Chen, Wu[3], Bo, Feng[1*]*

[1] Department of Metabolism and Endocrinology, Shanghai East Hospital, Tongji University School of Medicine, Shanghai, 200120, China

[2] Department of Cardiology, the First Affiliated Hospital of Chongqing Medical University, Chongqing, 400016, China

[3] Department of Cardiothoracic Surgery, the First Affiliated Hospital of Chongqing Medical University, Chongqing, 400016, China.

[#]These authors contributed equally.

[*]*Corresponding to*:

Bo, Feng, Department of Metabolism and Endocrinology, Shanghai East Hospital, Tongji University School of Medicine, Shanghai, 200120, China

E-mail:fengbo@medmail.com.cn

Qing-Chen, Wu, Department of Cardiothoracic Surgery, the First Affiliated Hospital of Chongqing Medical University, Chongqing, 400016, China

E-mail: qcwucq@163.com

Appendix 1: Table S1 Outcome data of clincial outcomes

StudyID	No. of patients	Primary outcomes						Secondary outcomes							
		POAF		AKI		MI		Mortality		MV (h)		ICU length of stay (d)		HLOS (d)	
		Statin	Control	Statin	Control	Statin	Control	Statin	Control	Statin	Control	Statin	Control	Statin	Control
Almansob et al 2012	68/64	0/68	1/64	NR	NR	NR	NR	NR	NR	NR	NR	NR	NR	NR	NR
Aydin et al 2015	30/30	5/30	13/30	NR	NR	1/30	0/30	1/30	0/30	5.50±1.30	5.80±1.60	2.30±1.05	2.40±1.50	6.90±2.60	7.40±3.50
Baran et al 2012	30/30	1/30	7/30	0/30	0/30	0/30	1/30	0/30	0/30	7.10±1.60	8.30±3.30	NR	NR	6.60±1.20	7.00±1.10
Berkan et al 2009	23/23	NR	NR	NR	NR	0/23	0/23	NR	NR	NR	NR	1.43±0.38	1.79±0.41	8.57±1.56	10.48±2.39
Billing et al 2016	308/307	115/308	103/307	10/308	8/307	NR	NR	4/308	1/307	NR	NR	3.00±0.83	3.00±0.83	NR	NR
Caoris et al 2008	21/22	5/21	8/22	NR	NR	NR	NR	NR	NR	NR	NR	NR	NR	NR	NR
Carascal et al 2016	47/43	20/47	13/43	3/47	0/43	2/47	0/43	NR	NR	16.9±4.44	8.13±8.14	3.34±4.75	2.79±2.49	10.22±7.22	8.28±3.28
Chello et al 2006	20/20	2/20	5/20	1/20	1/20	0/20	0/20	0/20	0/20	NR	NR	1.90±0.60	2.10±0.40	6.90±1.00	7.20±0.90
Chritanson et al 1999	40/37	NR	NR	3/40	8/37	0/40	5/37	NR	NR	NR	NR	2.10±0.80	2.00±0.90	11.60±3.20	11.50±2.20
Dehghani et al 2015	29/29	6/29	13/29	NR	NR	NR	NR	NR	NR	8.00±0	8.00±1.00	1.21±0.15	1.17±0.21	5.00±0	5.00±0
Ji et al 2009	71/69	10/71	23/69	NR	NR	0/71	1/69	NR	NR	13.60±4.90	14.80±5.5	2.02±0.36	2.09±0.44	12.40±2.10	12.80±2.20
Makuucdi et al 2005	152/151	NR	NR	NR	NR	1/152	4/151	6/152	11/151	NR	NR	NR	NR	NR	NR
Mannacio et al 2008	100/100	18/100	35/100	1/100	3/100	1/100	2/100	NR	NR	NR	NR	NR	NR	8.20±1.20	9.10±1.40
Melina et al 2009	315/317	94/315	106/317	NR	NR	NR	NR	NR	NR	NR	NR	NR	NR	NR	NR
Park et al 2016	100/100	42/100	50/100	21/100	26/100	NR	NR	1/100	0/100	16.58±2.71	16.00±3.45	2.00±0.33	2.00±0.67	13.00±2.00	14.00±3.00
Patti et al 2006	101/99	35/101	57/99	NR	NR	3/101	3/99	2/101	2/99	NR	NR	NR	NR	NR	NR
Prowle et al 2012	50/50	NR	NR	13/50	16/50	NR	NR	2/50	0/50	NR	NR	1.77±0.98	1.19±1.13	10.00±2.50	8.50±4.00
Song et al 2008	62/62	8/62	17/62	NR	NR	2/62	1/62	0/62	0/62	NR	NR	1.88±1.96	1.83±1.17	6.90±3.20	7.20±3.30
Sun et al 2011	49/51	9/49	21/51	NR	NR	0/49	1/51	NR	NR	19.40±5.30	21.30±6.40	2.90±0.61	2.98±0.68	13.60±1.60	14.20±2.10
Tamayo et al 2009	22/22	0/22	1/22	NR	NR	NR	NR	0/22	0/22	NR	NR	2.50±2.30	2.40±1.80	NR	NR
Vukovic et al 2011	29/28	4/29	11/28	NR	NR	1/29	1/28	0/29	0/28	14.30±8.60	17.20±4.70	NR	NR	NR	NR
Zheng et al 2016	960/962	149/960	117/962	237/960	186/962	37/960	41/962	3/960	1/962	NR	NR	1.75±0.69	1.71±0.67	7.00±0.67	7.00±0.67

POAF, Postoperative atrial fibrillation; AKI, Acute kindney injury; MV, Mechanical ventilation; ICU, Intensive care unit; HLOS, Hospital length of stay; MI, Myocardial infarction; NR: Not Reported;

Appendix 2: Table S2 Outcome of changes in CRP after surgery (mg/L)

**StudyID**		Almansob *et al* 2012	Aydin *et al* 2015	Baran *et al* 2012	Caoris *et al* 2008	Carascal *et al* 2016	Ji *et al* 2009	Mannacio *et al* 2008	Park *et al* 2016	Patti *et al* 2006	Song *et al* 2008	Sun *et al* 2011	Vukovic *et al* 2011	Zheng *et al* 2016
No. of patients		68/64	30/30	23/23	21/22	47/43	71/69	100/100	100/100	101/99	62/62	49/51	29/28	960/962
Day 1	Statin	64.55±4.41	63.00±17.60	NR	1.67±0.27	13.66±4.89	56.53±1.63	NR	75.2±12.43	NR	NR	56.38±1.74	8.11±3.32	NR
	Control	83.41±3.63	69.80±20.10	NR	1.98±0.12	13.76±5.29	71.70±2.40	NR	73.4±10.57	NR	NR	71.68±2.50	9.68±3.95	NR
Day 2	Statin	67.62±2.48	NR	NR	2.15±0.40	19.52±5.94	98.00±2.90	NR	NR	NR	NR	98.07±2.71	NR	NR
	Control	85.25±3.29	NR	NR	2.44±0.28	21.62±6.65	117.06±2.26	NR	NR	NR	NR	117.17±2.43	NR	NR
Day 3	Statin	50.66±1.91	NR	NR	NR	16.96±6.30	126.29±5.09	NR	NR	NR	NR	126.37±5.39	8.82±4.77	NR
	Control	63.35±2.60	NR	NR	NR	18.89±7.06	145.02±7.60	NR	NR	NR	NR	145.23±7.75	11.26±5.69	NR
Day 4	Statin	NR	NR	NR	NR	NR	112.47±4.00	NR	NR	NR	NR	112.43±4.12	NR	NR
	Control	NR	NR	NR	NR	NR	131.88±4.63	NR	NR	NR	NR	132.06±4.97	NR	NR
Day 5	Statin	23.52±0.99	NR	4.30±1.20	NR	NR	77.23±2.76	NR	NR	NR	NR	77.06±2.61	NR	50.90±27.89
	Control	35.77±2.26	NR	11.40±4.10	NR	NR	103.99±3.15	NR	NR	NR	NR	104.19±3.45	NR	57.20±31.02
Day 7	Statin	NR	33.70±13.70	NR	0.36±1.10	NR	24.91±1.10	NR	NR	NR	NR	24.89±1.20	NR	NR
	Control	NR	35.30±14.90	NR	0.82±0.21	NR	37.71±1.48	NR	NR	NR	NR	37.65±1.59	NR	NR
Peak	Statin	NR	NR	NR	NR	NR	126.50±22.30	15.40±2.50	NR	164.00±37.00	0.45±0.32	129.3±24.30	NR	NR
	Control	NR	NR	NR	NR	NR	145.20±31.60	17.20±3.40	NR	166.00±51.00	0.50±0.36	149.3±32.50	NR	NR

NR: Not Reported

Appendix 3: Table S3 Outcome data of changes in IL-6 after cardiac surgery (pg/ml)

StudyID	No of patients	≤12h		Day1		Day 2		Day 3	
		**Statin**	**Control**	**Statin**	**Control**	**Statin**	**Control**	**Statin group**	
Almansob *et al* 2012	68/64	18.14±3.68	33.20±3.05	NR	NR	NR	NR	NR	NR
Caoris *et al* 2008	21/22	255.20±42.00	282.00±48.70	159.50±58.50	251.20±53.00	81.90±31.50	194.20±56.30	NR	NR
Chello *et al* 2006	20/20	60.22±25.75	89.08±38.40	49.59±22.20	61.00±26.86	32.27±15.41	44.65±21.65	NR	NR
Tamayo *et al* 2009	22/22	139.44±48.62	146.01±91.71	98.52±42.86	104.75±31.63	56.69±31.39	47.47±20.62	NR	NR
Vukovic *et al* 2011	29/28	133.23±85.12	267.12±146.12	147.86±78.02	305.87±202.98	NR	NR	18.98±9.24	24.85±12.10

NR: Not Reported

Appendix 4: Table S4 Outcome data of changes in cTn after cardiac surgery (ng/L)

StudyID	No. of patients	4-6h		24h		72h		mean peak	
		Statin	Control	Statin	Control	Statin	Control	Statin	Control
Almansob *et al* 2012	68/64	0.88±0.56	1.35±1.56	0.59±0.44	0.91±1.08	0.36±0.29	0.61±0.74	NR	NR
Baran *et al* 2012	30/30	1.40±1.80	2.30±2.20	NR	NR	NR	NR	NR	NR
Berkan *et al* 2009	23/23	NR	NR	61.10±2.08	42.11±0.93	NR	NR	NR	NR
Carascal *et al* 2016	47/43	1.23±1.77	0.66±0.67	1.03±1.57	0.49±0.64	0.69±1.07	0.38±0.48	NR	NR
Mannacio *et al* 2008	100/100	NR	NR	NR	NR	NR	NR	0.16±0.15	0.32±0.26
Vukovic *et al* 2011	29/28	2.53±1.56	2.65±2.22	1.43±1.95	2.56±2.22	0.86±0.88	1.59±1.10	NR	NR
Zheng *et al* 2016	960/962	NR	NR	2.60±0.10	2.60±0.10	NR	NR	NR	NR

NR: Not Reported

Appendix 5 Figure S1 Quality assessment

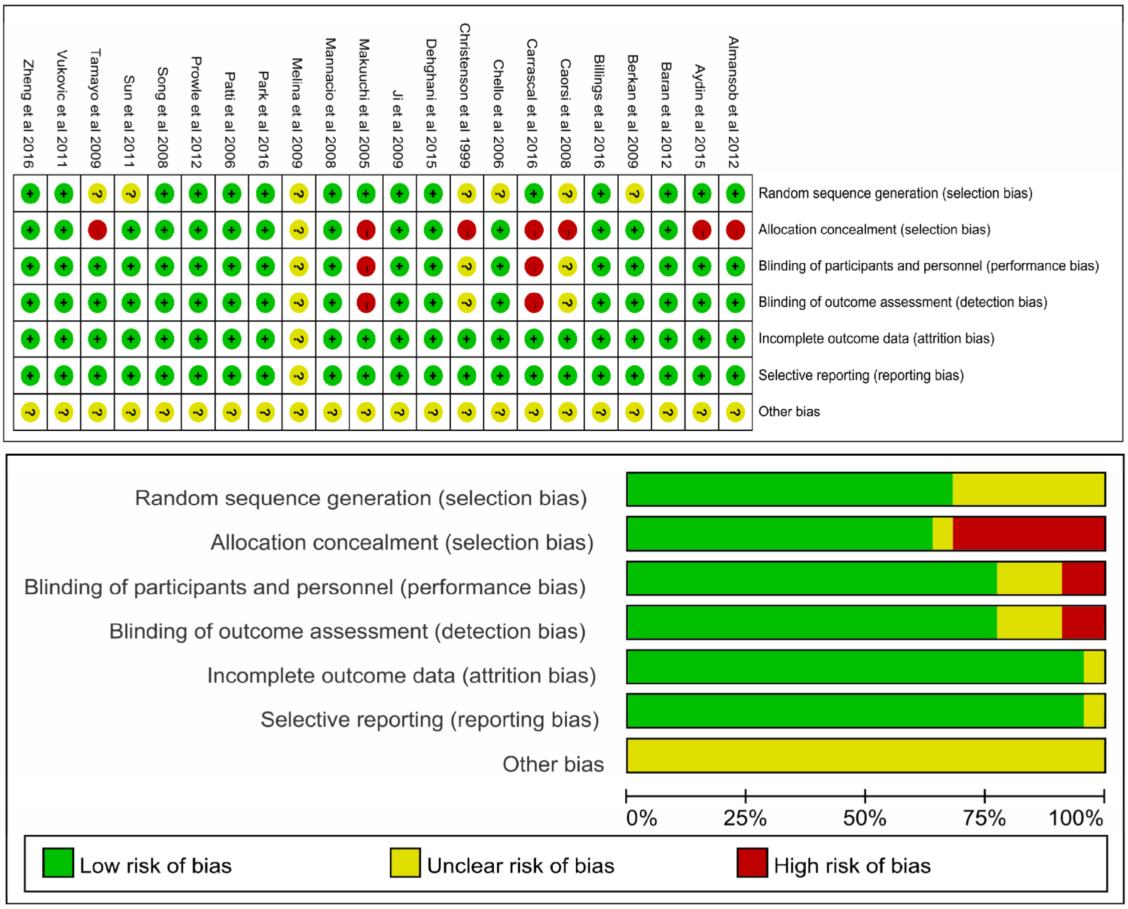

Appendix 6: Search strategy

#1 "hydroxymethylglutaryl-coa reductase inhibitors"[MeSH Terms]

#2 Statin [All Fields]

#3 #1 OR #2

#4 "cardiovascular surgical procedures"[All Fields]

#5 "cardiac surgical procedures"[All Fields]

#6 "cardiopulmonary bypass"[MeSH Terms]

#7 "cardiopulmonary bypass"[All Fields])

#8 "coronary artery bypass"[MeSH Terms]

#9 "coronary artery bypass"[All Fields]

#10 "coronary artery bypass surgery"[All Fields])

#11 "CABG"[All Fields]

#12 "valve surgery"[All Fields]

#13 "thoracic surgery"[MeSH Terms]

#14 "thoracic surgery"[All Fields]

#15 "cardiac surgery"[All Fields]

#16 "cardiac surgical procedures"[MeSH Terms]

#17 "cardiac surgical procedures"[All Fields]

#18 "thoracic surgery"[MeSH Terms]

#19 "thoracic surgery"[All Fields]

#20 "heart surgery"[All Fields]

#21 "cardiac surgical procedures"[MeSH Terms]

#22 "cardiac surgical procedures"[All Fields]

#23 OR/4-22

#24 #3 AND #23

#25 “RCT”

#26 #24 AND #25

RCT= (randomized controlled trial[pt]OR randomized controlled trials[mh]OR random allocation[mh] OR random allocat* [tw] OR randomly allocat* [tw] OR double-blind method[mh] OR single-blind method[mh] OR double blind* [tw] OR single blind[tw] OR triple blind*[tw]OR clinical trial [pt] OR clinical trials[mh])
